# Combined clinical and laboratory diagnostics of neurodegenerative disorders

**DOI:** 10.1192/j.eurpsy.2023.1035

**Published:** 2023-07-19

**Authors:** A. Sidenkova, V. Litvinenko

**Affiliations:** Psychiatry, Ural State Medical University, Yekaterinburg, Russian Federation

## Abstract

**Introduction:**

Alzheimer’s disease (AD) is a neurodegenerative pathology that develops mainly in elderly and senile people.

**Objectives:**

The aim of the study was a comparative analysis of the results of neuropsychological tests with the indicators of the study of oral fluid and buccal epithelium cytograms in patients with Alzheimer’s disease.

**Methods:**

In the main group of 12 patients with Alzheimer’s disease, m=76.25±4.89. There were 12 cognitively healthy people in the control group. The average MMSE score among the observations of the main group was 13.42±3.63. The ADAS-COG scale was used to detail the impaired cognitive functions. The concomitant pathology is compensated. The content of BDNF, TNF-α, IL1RA, IL-6, and IL-8 was determined in the oral fluid and in the blood serum. The concentration of salivary and serum biomarkers was determined by multiparametric fluorescence analysis with magnetic microspheres. Micronuclei, karyopycnosis, karyorexis, and karyolysis were determined in the cellular structures of the buccal epithelium. The material for cytological examination was collected from the inner surface of the cheek.

**Results:**

When analyzing buccal cytograms, attention was drawn to a pronounced increase in the number of cells with micronuclei in patients with AD to 1.8%; in the control group, the median was 0.1% (p<0.05). A direct correlation was established between the number of binuclear cells and the level of BDNF in the blood serum (r=0.646; p=0.03) in patients with AD. It is also important to note that the level of serum BDNF had a significant direct correlation with immediate memory, and the concentration of salivary BDNF correlates with the parameter of naming objects.

**Conclusions:**

Correlations between amnesia, speech disorders, praxis, gnosis and pathology of the oral fluid and buccal epithelium, especially with the severity of karyopycnosis and karyorexis, have been established, indicating a direct correlation between the neurodegenerative process pathogenetically associated with Alzheimer’s disease and the processes of systemic inflammation and degeneration of the buccal epithelium.

**Disclosure of Interest:**

None Declared

